# Artificial Intelligence Tools That Improve Medication Adherence in Patients With Chronic Noncommunicable Diseases: An Updated Review

**DOI:** 10.7759/cureus.83132

**Published:** 2025-04-28

**Authors:** Esteban Zavaleta-Monestel, Luis Carlos Monge Bogantes, Silvia Chavarría-Rodríguez, Sebastián Arguedas-Chacón, Natalia Bastos-Soto, Jorge Villalobos-Madriz

**Affiliations:** 1 Pharmacy, Hospital Clínica Bíblica, San José, CRI; 2 Faculty of Pharmacy, Universidad Latina de Costa Rica, San José, CRI; 3 Research, Hospital Clínica Bíblica, San Jose, CRI

**Keywords:** artificial intelligence(ai), chronic disease managment, digital health tools, medication adherence strategies, pharmaceutical care

## Abstract

This systematic review analyzes the use of artificial intelligence (AI) tools to improve medication adherence in patients with chronic non-communicable diseases, with a specific focus on their implementation in pharmaceutical care. Medication non-adherence remains a major barrier to effective chronic disease management, contributing to poor clinical outcomes and rising healthcare costs. AI offers promising, data-driven approaches to address this challenge through tools such as conversational agents, mobile applications, smart devices, and adherence classifiers. These tools enhance patient monitoring, education, and engagement, enabling personalized interventions to promote consistent medication use. The 26 included studies were evaluated based on their methodology, type of AI tool, healthcare setting, and reported impact on adherence outcomes. Most reported improvements in adherence, though variation in assessment methods limits comparability. Ethical, legal, and accessibility issues remain key challenges to wider adoption. Overall, AI represents a valuable and emerging strategy for supporting adherence and optimizing pharmaceutical care in chronic disease management.

## Introduction and background

Medication adherence remains one of the major challenges in the management of chronic non-communicable diseases, contributing to disease progression, increased morbidity and mortality, and elevated healthcare costs. Pharmaceutical care, defined as the set of actions aimed at ensuring comprehensive, integrated, and continuous attention to the medication needs of the population, plays a crucial role in addressing this challenge [1]. Collaborating with physicians and other healthcare professionals, pharmacists are central to improving treatment adherence and achieving better health outcomes. Although the implementation of pharmaceutical care varies globally, this review includes studies from multiple health systems, without restriction to a particular country. Moreover, the integration of pharmacists in adherence programs remains limited in some regions, such as parts of Latin America, where their participation is not fully embedded in national strategies [1].

In parallel, artificial intelligence (AI) has emerged as a transformative force in healthcare over the past five decades, enabling new approaches to disease management, data analysis, and personalized care [2]. AI involves logical algorithms capable of learning from data and making autonomous decisions based on generalizable rules [3,4]. Within the pharmaceutical sector, AI has been increasingly adopted to support innovation and address complex healthcare needs [5].

Since barriers to medication adherence are multifactorial, including behavioral, social, and systemic factors, solutions must also be multifaceted [2]. AI-based tools offer a promising avenue to address these complexities by enhancing patient monitoring, predicting adherence risks, personalizing interventions, and facilitating communication between healthcare providers and patients [6].

This article presents a systematic review of the scientific literature evaluating AI tools designed to improve medication adherence in patients with chronic non-communicable diseases. The review focuses on their implementation in pharmaceutical care, the specific role of pharmacists in their application, and the broader ethical, legal, and accessibility implications. Furthermore, this review classifies AI interventions into four main categories, conversational agents, mobile applications, smart devices, and adherence classifiers, and critically examines their impact on adherence outcomes.

By systematically analyzing the available evidence, this review aims to provide healthcare professionals, researchers, and policymakers with a comprehensive understanding of the current capabilities, benefits, and limitations of AI-driven adherence interventions in pharmaceutical practice.

## Review

Methods

This study was conducted through a systematic review of the scientific literature focusing on the use of AI tools to enhance medication adherence in patients with chronic non-communicable diseases. A structured search strategy was employed across PubMed, ScienceDirect, and Google Scholar, using a three-tiered approach to identify relevant publications. The primary search combined the terms “artificial intelligence,” “medication adherence,” and “chronic diseases,” while the secondary and tertiary searches focused on “artificial intelligence,” “medication adherence,” and “benefits,” as well as “limitations,” respectively. Boolean operators (AND, OR, NOT) were consistently applied to refine the search results across all databases.

Filters were set to include publications in English or Spanish, with full-text access (whether open access or subscription-based), encompassing primary research articles, bibliographic reviews, and systematic reviews published between January 2017 and January 2025.

Inclusion Criteria

The review included studies involving pharmacists (clinical or hospital), physicians, and computer scientists using AI tools within public or private medical centers, as well as universities. Eligible studies had to focus on the role and contribution of AI in medication management, error prevention, or the improvement of therapeutic adherence. Studies were required to have been conducted in healthcare settings such as hospitals, clinics, community pharmacies, or primary care centers, within both the public and private sectors, and across diverse geographical regions. Only full-text studies published in English or Spanish within the specified time frame, and that assessed adherence over a period of at least six months, were included. The review accepted experimental and quasi-experimental studies, including randomized and non-randomized controlled trials, as well as analytical and descriptive observational studies that met established methodological quality standards. Literature reviews, systematic reviews, and meta-analyses meeting the inclusion criteria were also considered.

Exclusion Criteria

Studies were excluded if they had a sample size of fewer than 30 participants or if they did not directly address the use of AI tools in pharmaceutical practice, specifically regarding medication management and therapeutic adherence. No exclusions were made based on culture, race, ethnicity, or gender. Any discrepancies during the study selection process were resolved by consensus among the reviewers or, if necessary, with the intervention of a third reviewer.

Data Extraction and Synthesis

Data were extracted using a standardized template, collecting details such as study design, AI intervention type, healthcare setting, country, population characteristics, adherence assessment method, and primary outcomes. The information was synthesized narratively and presented in figures and tables to facilitate interpretation. Studies were grouped according to the four main categories of AI-based adherence interventions identified in the literature: conversational agents, mobile applications, smart devices, and adherence classifiers. Within each category, results were compared, and contextual factors were discussed to highlight the impact and variability of outcomes.

Quality Assessment

When applicable, the methodological quality of the included studies was assessed using validated tools such as the CASP checklist for qualitative research or the ROBINS-I tool for non-randomized studies. However, no studies were excluded solely based on their quality assessment results.

Declaration of AI Tool Usage

ChatGPT was used exclusively to enhance the clarity, structure, and flow of the manuscript. No artificial intelligence tools were used for data analysis, interpretation of results, or the generation of scientific content beyond linguistic refinement.

Results

A total of 7,931 articles were identified across PubMed, ScienceDirect, and Google Scholar through the structured search strategy. After removing 2,184 duplicates, a total of 5,747 unique records were retained. Of these, 3,338 articles were excluded during the initial screening based on their titles and abstracts due to a lack of relevance to the research question. Irrelevant articles included studies that did not focus on artificial intelligence applications, those unrelated to pharmaceutical practice, or those addressing general aspects of adherence without AI components.

**Figure 1:**
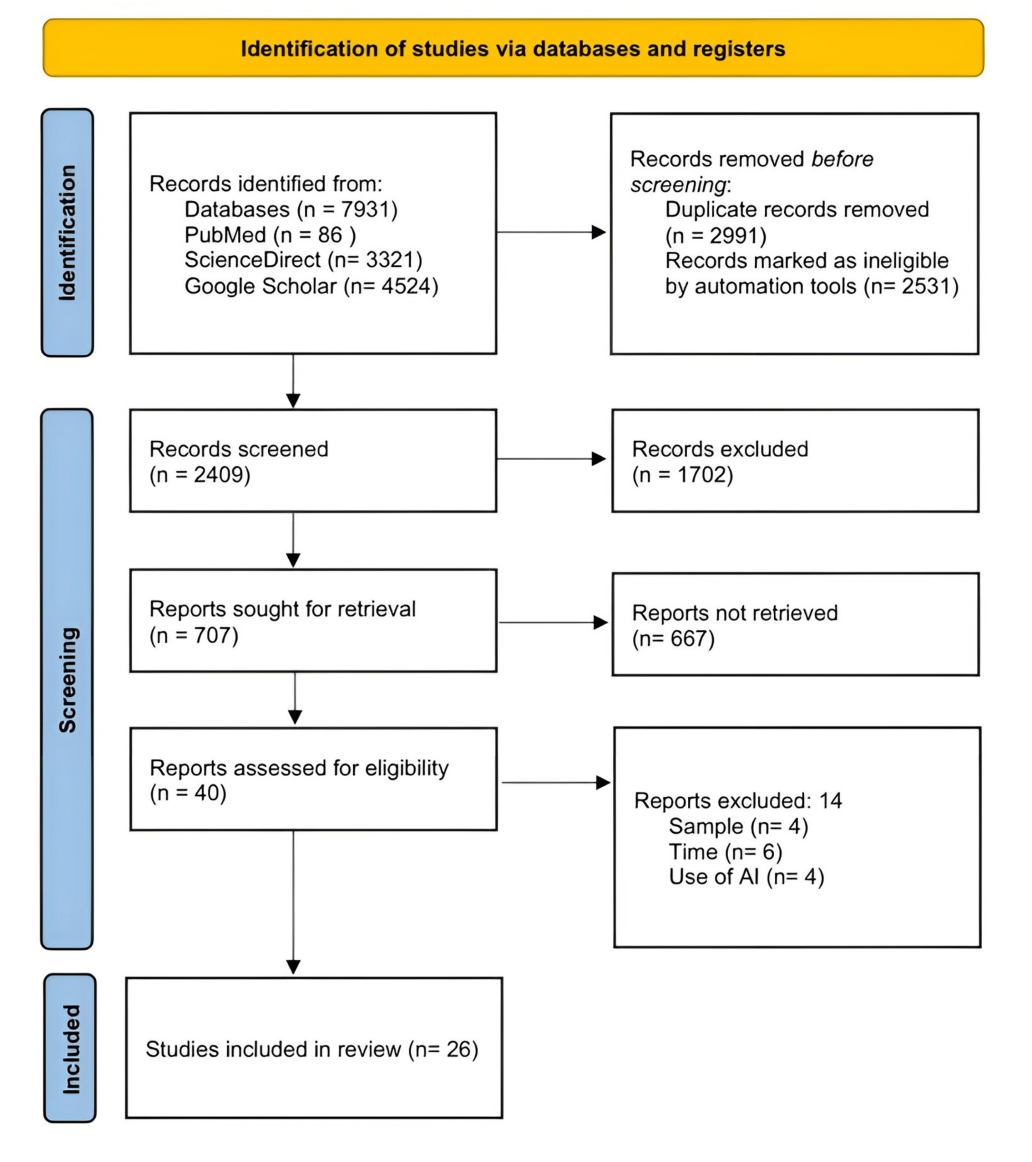
PRISMA flow diagram PRISMA: Preferred Reporting Items for Systematic Reviews and Meta-Analyses

The remaining 2,409 articles underwent full-text assessment. During this phase, 2,383 records were excluded for not meeting the predefined inclusion criteria. Reasons for exclusion included a sample size smaller than 30 participants, lack of a clear focus on AI-driven interventions to improve medication adherence, absence of pharmacist involvement, or insufficient methodological detail. Additionally, 22 articles could not be retrieved in full text despite institutional access and database search efforts; these were also excluded from the synthesis. Ultimately, 26 studies were included in the qualitative synthesis. The flow of the study selection process is illustrated in Figure 1, following the Preferred Reporting Items for Systematic Reviews and Meta-Analyses (PRISMA) 2020 guidelines.

Table 1 provides an overview of the included studies, which comprised a diverse array of designs including literature reviews, systematic reviews, clinical trials, and observational studies. These works collectively examine the application of AI in pharmaceutical care, with a focus on enhancing medication adherence, supporting clinical decision-making, and improving patient outcomes. Several studies addressed technological innovation and implementation challenges, while others examined ethical, legal, and regulatory aspects essential for the integration of reliable AI systems. Table 1 summarizes the study design, population, type of AI intervention, adherence outcomes, and key conclusions of each included article.

**Table 1:** Characteristics of the included studies

Study	Type of study	Summary
Chalasani et al. [2]	Literature review	Reviews AI applications in pharmaceutical practice, emphasizing the need for clear implementation guidelines
Foley et al. [3]	Systematic review	Examines medication non-adherence in multimorbid patients, identifying prevalence and predictors
González-Pérez et al. [4]	Systematic review and meta-analysis	Explores AI’s role in health sciences, with a focus on pharmaceutical care
Vora et al. [5]	Literature review	Highlights current pharmaceutical challenges and limitations of AI adoption
Babel et al. [6]	Literature review	Assesses AI solutions to improve adherence in patients with non-communicable diseases
Kardas [7]	Literature review	Investigates whether innovative technologies can address medication non-adherence
Schachner et al. [8]	Systematic review	Notes limited evidence on chatbot use for chronic disease management; most remain prototypes
Chang et al. [9]	Evaluation and development	Medication recognition systems reduce administration errors and enhance treatment adherence
Peng et al. [10]	Systematic review	Mobile apps are linked to improved adherence in chronic disease patients
Morawski et al. [11]	Randomized clinical trial	Medi-SAFE app improved self-reported adherence
Arshed et al. [12]	Randomized clinical trial	mHealth interventions in Washington improved hypertension treatment adherence
Horne et al. [13]	Randomized clinical trial	Behavioral nudges increased statin adherence in cardiac patients
Pleasants et al. [14]	Literature review	AI tools improve inhaled therapy adherence and administration
Li et al. [15]	Pilot clinical trial	Smartwatches enhanced adherence and symptom control; enabled timely interventions
Lee and Youm [16]	Evaluation and development	Medication management systems using wearables showed high accuracy and telemedicine potential
Xie et al. [17]	Literature review	Describes integration of blockchain and AI wearables; notes associated challenges
Rajput et al. [18]	Literature review	Pulsatile drug delivery systems optimize dosage timing, supporting adherence
Worral et al. [19]	Retrospective study multicenter quasi-experimental	Pharmacist- and AI-driven services improve adherence via technology and connectivity
Kanyongo et al. [20]	Observational study	AI models detect non-adherence and enable targeted, efficient clinical interventions
Korb-Savoldelli et al. [21]	Observational study	Tools support patient prioritization and referral based on individualized responses
Oh et al. [22]	Cost-effectiveness	Pharmacist- and AI-led interventions are cost-effective in primary care
Días-Rodríguez [23]	Literature review	Analyzed the principles, pillars, and requirements that AI systems must meet to be considered reliable.
Huang et al. [24]	Literature review	Outlines principles and requirements for trustworthy AI systems
Zhang and Zhang [25]	Literature review	Analyzes ethical challenges: bias, opacity, safety, and accountability
Gerke et al. [26]	Literature review	Reviews U.S. and European strategies addressing legal and ethical issues in healthcare AI
Kerasidou [27]	Literature review	Stresses the importance of empathy, compassion, and trust in AI integration

Discussion

**Figure 2:**
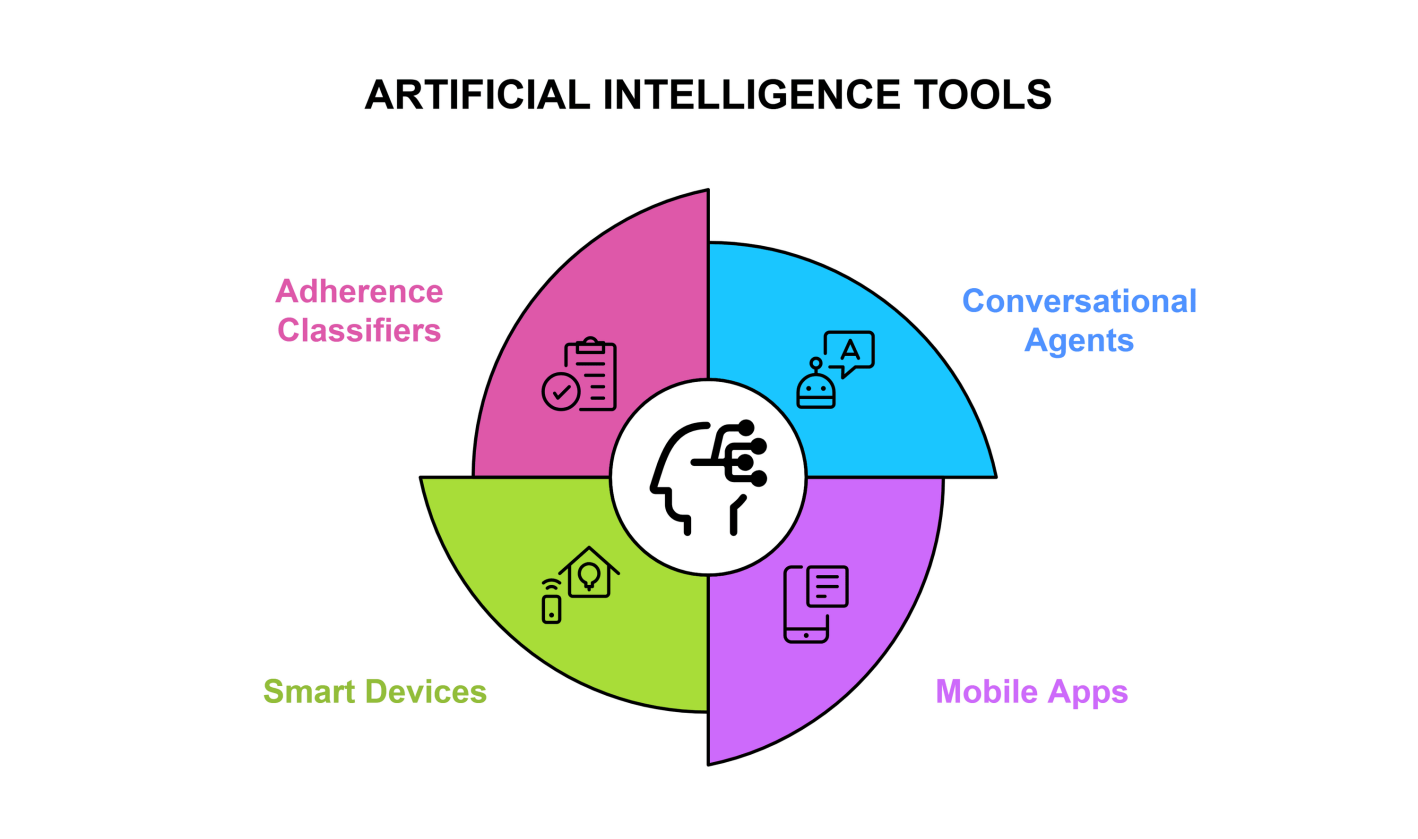
Artificial intelligence tools for improving medication adherence

This section presents various AI-driven solutions (Figure 2) designed to enhance medication adherence in patients with chronic non-communicable diseases. These include mobile applications, conversational agents, smart devices, and adherence classification tools. Collectively, these strategies aim to optimize therapeutic compliance, facilitate treatment monitoring, and ultimately improve health outcomes. Their application in pharmaceutical care has shown promise in enhancing clinical decision-making, promoting personalized patient engagement, and supporting system-level efficiencies.

Conversational Agents

Conversational agents, or chatbots, are increasingly used in chronic disease management through natural language processing (NLP) to interact with patients, provide personalized education, and send timely medication reminders. These tools are capable of remote monitoring and data integration with mobile platforms [8]. For instance, ST-Med-Box employs deep learning for drug recognition to assist with polypharmacy management. It provides reminders, access to medical information, and medication history tracking, achieving a drug identification accuracy of 96.6%, thereby improving safety and adherence [9].

Mobile Applications

Mobile health applications use data analytics and machine learning to predict adherence behaviors and personalize interventions. While some apps, such as MediSAFE, have shown modest improvements in self-reported adherence in hypertensive patients without corresponding improvements in clinical outcomes like blood pressure control [13], others have demonstrated greater impact. For example, the mHealth application based on the Health Belief Model, integrated with WhatsApp, led to statistically significant improvements in adherence and blood pressure reduction [12]. The ENCOURAGE trial further validated the use of AI-driven behavioral nudges to enhance statin adherence by tailoring reminders to individual psychographic profiles [13].

Smart Devices

Smart technologies, particularly in the form of digital inhalers and wearable devices, have shown potential in chronic respiratory and allergic conditions. Devices such as InHandPlus and AI-based smartwatch systems accurately detect drug administration events, facilitating real-time adherence tracking [15,16]. Furthermore, the integration of AI with blockchain and Pulsatile Drug Delivery Systems (PDDS) introduces opportunities for secure data management and personalized, on-demand medication delivery, especially relevant in polypharmacy and comorbid patients [17,18].

Adherence Classifiers

AI-based classifiers are used to stratify patients by adherence risk using prescription claims, medication gaps, and patient-reported outcome measures (PROMs). These systems enable pharmacists to prioritize patients for interventions, improve the allocation of healthcare resources, and prevent unnecessary efforts directed at already adherent individuals [19-21]. They are also capable of identifying behavioral predictors through machine learning algorithms, offering a powerful tool for preemptive intervention design [20].

Benefits of AI Tools

Across the studies reviewed, improved adherence emerged as a consistent and central outcome. Among the 20 articles focusing on AI’s clinical applications, eight directly measured adherence improvement as the primary outcome. The tools contributed not only to better pharmacotherapeutic follow-up but also to patient empowerment through education, reminders, and support systems. Table 2 summarizes the studies reporting these outcomes.

**Table 2:** Summary of the relevant results of artificial intelligence tools

Author	Type of study	Location	Sample	AI tool	Tool classification	Results
Morawski et al. (11)	Randomized clinical trial	United States	411	Medisafe mobile application	Predictive analytics	Improved self-reported medication adherence in patients with hypertension
Arshed et al. (12)	Randomized clinical trial	Pakistan	439	mhealth multiple AI package	Narrow AI	Improved medication adherence and therapeutic outcomes in patients with hypertension
Horne et al. (13)	Randomized controlled trial	United States	182	Behavioral nudges	Machine and reinforcement learning	Increase statin adherence by improving health through patient-guided choices
Li et al. (15)	Pilot clinical trial	China	60	Smart Watch InHandPlus	Machine learning	Improved medication adherence and symptom control in allergic rhinitis
Worral et al. (19)	Multicenter quasi-experimental retrospective study	United States	10,477	Adherence program	Predictive analysis	It allowed classifying patients according to their risk of adherence, improved medication adherence in patients with hypertension, cholesterol, and diabetes
Kanyongo et al. (20)	Observational study	Zimbabwe	8141	Classification tool	Machine learning	It allows classifying patients according to their adherence, enabling the prioritization of interventions
Korb-Savoldelli et al. (21)	Observational study	France	218	Patient-reported outcome measures	Machine learning	Enabled patient classification based on adherence, allowing for intervention prioritization
Oh et al. (22)	Cost-effectiveness	South Korea	1004	Monitoring service	Machine learning	The intervention was most costly but more effective

Optimization of Patient Selection

AI tools were instrumental in improving patient selection for pharmaceutical interventions, enabling a more precise and evidence-based allocation of care. Several programs successfully segmented non-adherent patients and prioritized resources for those with the greatest potential benefit [19-24]. This approach reduces intervention costs and supports sustainable implementation of adherence strategies in health systems under pressure.

Cost-Benefit Analysis

The economic evaluations reported in the literature indicate that AI-based adherence programs may lead to cost savings despite higher initial investments. In Texas, a program reported up to 32% reductions in costs related to chronic disease management [20]. A cost-effectiveness study in South Korea showed that an AI-based intervention improved quality-adjusted life years (QALYs) with an acceptable incremental cost-effectiveness ratio of $5,556/QALY gained [22]. These findings reinforce the idea that AI implementation, if properly designed, can be both clinically and economically sustainable.

Limitations of AI Tools

Despite their promise, AI tools face several limitations. Key among these is the “black box” nature of many machine learning systems, which limits transparency and undermines trust and clinical accountability [24,25]. Legal, ethical, and technical safeguards must be integrated into development and implementation. These include adherence to bioethical principles, such as non-maleficence, beneficence, and respect for autonomy, as well as equitable access and cultural adaptability [24,26,27]. Current models frequently lack mechanisms for formal informed consent, relying instead on user agreements that may not guarantee meaningful autonomy or privacy protection [25].

Critical Gaps and Future Directions

While evidence supports the effectiveness of AI in enhancing adherence, many studies are still at the pilot or observational stage, limiting generalizability. Randomized controlled trials remain scarce, and heterogeneity in adherence definitions, outcome measures, and patient populations complicates comparison. Additionally, few interventions were developed or tested in low- and middle-income countries, highlighting a critical gap in global applicability.

Future research should focus on developing explainable AI models, incorporating co-design with pharmacists and patients, and ensuring ethical frameworks that support informed participation. Moreover, health policy should encourage reimbursement structures and regulatory pathways that facilitate the integration of trustworthy AI into routine pharmaceutical practice.

## Conclusions

AI has demonstrated measurable benefits in improving medication adherence in patients with chronic diseases, particularly through the use of conversational agents, mobile applications, smart devices, and risk-based adherence classifiers. These tools support personalized care, strengthen pharmacotherapeutic follow-up, and optimize resource allocation in pharmaceutical practice. However, their successful integration depends on addressing ethical concerns, data transparency, and equitable access. Future efforts should focus on scalable, explainable, and patient-centered AI systems that are ethically governed and clinically validated. Integrating such tools into routine pharmaceutical care may represent a transformative step toward improving long-term health outcomes and medication use in chronic disease management.
